# Correction: A benzylic linker promotes methyltransferase catalyzed norbornene transfer for rapid bioorthogonal tetrazine ligation

**DOI:** 10.1039/c7sc90073b

**Published:** 2017-11-16

**Authors:** F. Muttach, N. Muthmann, D. Reichert, L. Anhäuser, A. Rentmeister

**Affiliations:** a University of Münster , Department of Chemistry , Institute of Biochemistry , Wilhelm-Klemm-Str. 2 , 48149 Münster , Germany; b Cells-in-Motion Cluster of Excellence (EXC1003-CiM) , University of Münster , Germany . Email: a.rentmeister@uni-muenster.de

## Abstract

Correction for ‘A benzylic linker promotes methyltransferase catalyzed norbornene transfer for rapid bioorthogonal tetrazine ligation’ by F. Muttach *et al.*, *Chem. Sci.*, 2017, DOI: ; 10.1039/c7sc03631k.



## 

The authors regret that Fig. 4 is incorrect in the original manuscript. In Fig. 4c the chemical structure and mass spectrum of the norbornene-modified adenosine was shown instead of the 2′-deoxyadenosine. The correct figure and caption are displayed below.

**Fig. 1:**
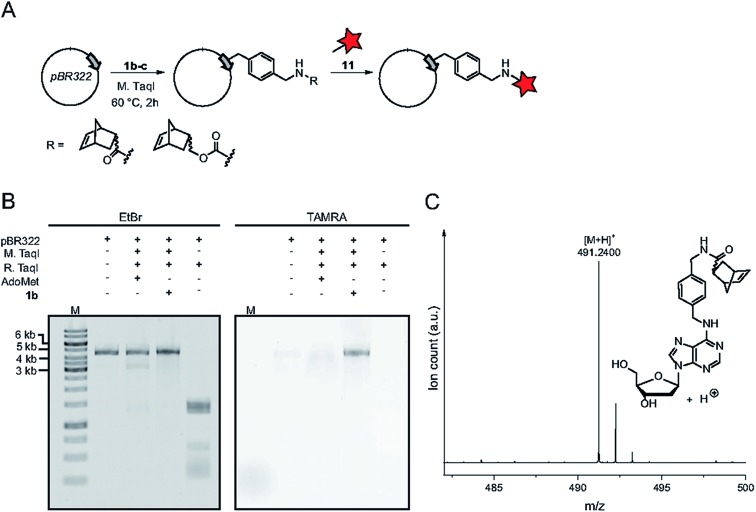
Norbornene modification of pBR322 plasmid DNA using the *N*^6^-adenine MTase M. TaqI. (A) Scheme for the functionalization of plasmid DNA using norbornene-modified AdoMet analog **1b**. (B) Fluorescence labeling of plasmid DNA *via* norbornene-modification followed by labeling with TAMRA-tetrazine and linearization of the plasmid using BamHI. Bands were resolved on a 1% agarose gel (100 V, 50 min), the gel was stained using ethidium bromide and scanned on a Typhoon FLA9500 laser scanner. (C) Mass spectrometric analysis of *N*^6^-norbornene-modified oligonucleotides. A DNA oligonucleotide was subjected to enzymatic norbornene-modification, followed by digestion using nuclease P1 and dephosphorylation using FastAP (ThermoFisher Scientific). Expected mass for C_26_H_31_N_6_O_4_^+^ = 491.2401 [M + H]^+^, found: 491.2400. M: GeneRuler 1 kb DNA ladder (ThermoFisher).

The Royal Society of Chemistry apologises for these errors and any consequent inconvenience to authors and readers.

